# Advances in Breast Cancer Diagnosis and Treatment in Africa

**DOI:** 10.3390/biology15181645

**Published:** 2026-09-17

**Authors:** Anja Piso, Eman Teer, Unathi Ramashala, Claudia du Plessis, Anne Elizabeth Mercier, Anna-Mart Engelbrecht, Iman van den Bout

**Affiliations:** 1 Department of Physiology, Faculty of Health Sciences, University of Pretoria, Pretoria 0031, South Africa; u19015756@tuks.co.za (A.P.); unathi.ramashala@tuks.co.za (U.R.); u20429992@tuks.co.za (C.d.P.); joji.mercier@up.ac.za (A.E.M.); 2 Department of Physiological Sciences, Faculty of Science, Stellenbosch University, Stellenbosch 7600, South Africa; emanteer@sun.ac.za (E.T.); ame@sun.ac.za (A.-M.E.)

**Keywords:** breast cancer, Africa, precision oncology, treatment, cancer biology, diagnostics

## Abstract

Breast cancer remains a significant cause of cancer-related mortality in Africa, where late-stage diagnosis, limited access to specialised care, and underrepresentation in biomedical research continue to impede improvements in patient outcomes. At the same time, rapid advances in tumour biology, molecular diagnostics, and precision oncology are fundamentally reshaping our understanding of breast cancer globally. This review examines recent developments in breast cancer clinical research, highlighting how these developments are shaping the future of diagnostics and targeted therapeutics. We further discuss emerging technologies, including multi-omics, liquid biopsy, and artificial intelligence, and consider their potential to advance precision oncology. Importantly, we evaluate these advances within the African context, where limited molecular data, restricted access to diagnostic infrastructure, and resource constraints continue to challenge clinical implementation. Improving outcomes across the continent will require more than the adoption of emerging technologies; it will depend on generating African-relevant biological evidence, expanding representation in genomic research, and integrating molecular, clinical, and environmental data to develop equitable, context-specific precision medicine strategies. Such an approach offers the potential to transform breast cancer management while reducing longstanding disparities in cancer care across Africa.

## Simple Summary

Breast cancer is the most diagnosed cancer in women worldwide and is becoming an increasing health challenge across Africa. Although many scientific advances have improved how breast cancer is detected and treated, women in many African countries continue to experience poorer outcomes because cancers are often diagnosed at a later stage and access to specialised care, molecular testing, and modern therapies remains limited. At the same time, African populations are underrepresented in much of the research that guides current clinical practice, making it difficult to ensure that new technologies are equally effective for all patients. This review highlights recent advances in breast cancer diagnosis, and treatment, including precision medicine, artificial intelligence, and discusses how these developments could improve cancer care in Africa. We also explore the challenges that must be addressed to ensure equitable access to embed these new technologies in clinical practice. By increasing African participation in research and developing healthcare solutions that are tailored to local needs, these advances have the potential to improve earlier diagnosis, support more personalised treatment, and ultimately reduce breast cancer deaths across the continent.

## 1. Introduction

**Figure 1:**
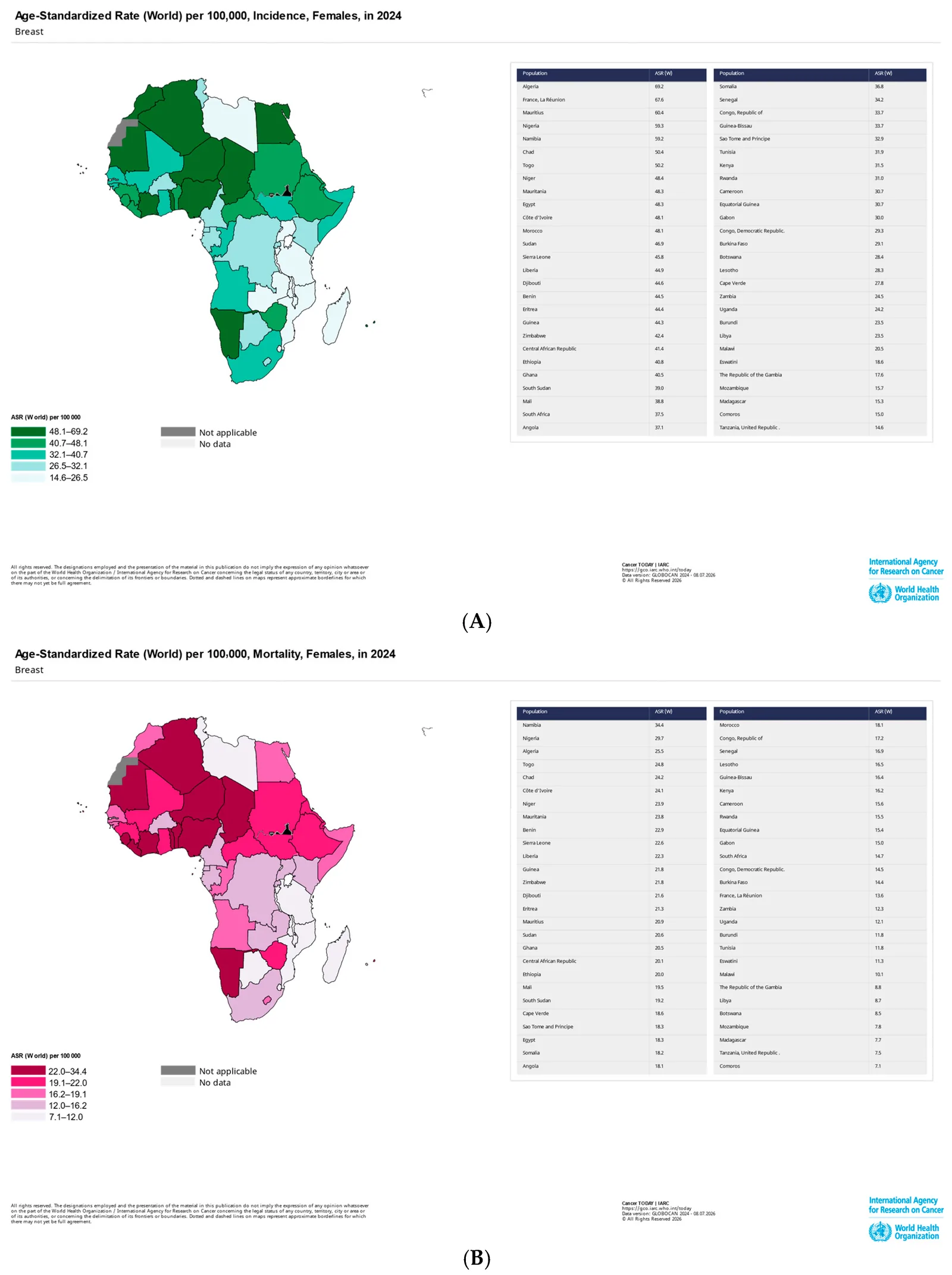
Geographic distribution of breast cancer burden among females across Africa in 2024. Age-standardised rates (ASR, World standard) per 100,000 are shown for (**A**) incidence and (**B**) mortality, as estimated by GLOBOCAN 2024 [2]. Algeria recorded the highest incidence rate (ASR 69.2), while Namibia recorded the highest mortality rate (ASR 34.4). Notable disparities are observed across sub-regions, with North and West African countries generally reporting higher incidence rates relative to East and Southern African nations. Source: Cancer Today, IARC/WHO (https://gco.iarc.who.int/today; accessed on 8 July 2026).

Breast cancer is now the most frequently diagnosed cancer among women worldwide, accounting for a rapidly increasing proportion of the global cancer burden. Although captured statistics of African countries document a lower incidence than many high-income countries (HICs), a disproportionately high mortality is also reported, highlighting inequities in early detection, access to treatment, and cancer care infrastructure (Figure 1) [1,2,3].

The geographic distribution of breast cancer burden across Africa is heterogeneous, reflecting variations in urbanisation, lifestyle factors, healthcare infrastructure, and data collection capacity. It is important to note here that Africa is not a single hegemony; with the highest level of genetic diversity globally, as well as varied environments and cultures, it is assumed that there will also be inherent differences in cancer demographics between regions. North African countries report higher incidence rates (40–60 per 100,000), approaching those of Southern Europe, while sub-Saharan regions show lower but rapidly increasing rates [4]. Urban populations experience higher incidence than rural areas, attributed to reproductive factors (delayed childbearing, reduced breastfeeding), dietary changes, and increased obesity prevalence, although the impact of divergent reporting on cancer incidence can impact these comparisons [5,6]. However, rural populations face greater mortality due to limited access to diagnostic and treatment facilities [7].

Unlike many HICs, where population-based screening programmes facilitate early detection, most African women present with locally advanced or metastatic disease (Stage III–IV) [8]. The reasons for delayed presentation are multifactorial, including limited breast cancer awareness, cultural beliefs, financial constraints, long travel distances to specialised centres, and weaknesses in referral pathways. Consequently, many patients require more aggressive multimodal treatment, including radical surgery, radiation, systemic chemotherapy, and palliative interventions [9,10]. Breast cancer also presents with disparities that are, at least in part, due to biological heterogeneity between different groups. This includes phenomena such as the much younger age at first diagnosis of black African women versus white women in South Africa, with median ages at diagnosis ranging from 40 to 50 years compared to 60 to 65, respectively [11,12]. A second biological difference includes the higher prevalence of more aggressive cancer subtypes. Triple-negative breast cancer (TNBC), an aggressive subtype lacking oestrogen receptor, progesterone receptor, or increased HER2 expression, occurs at substantially higher frequencies in African women (25–40%) compared to European and North American populations (10–20%) [13,14].

**Figure 2:**
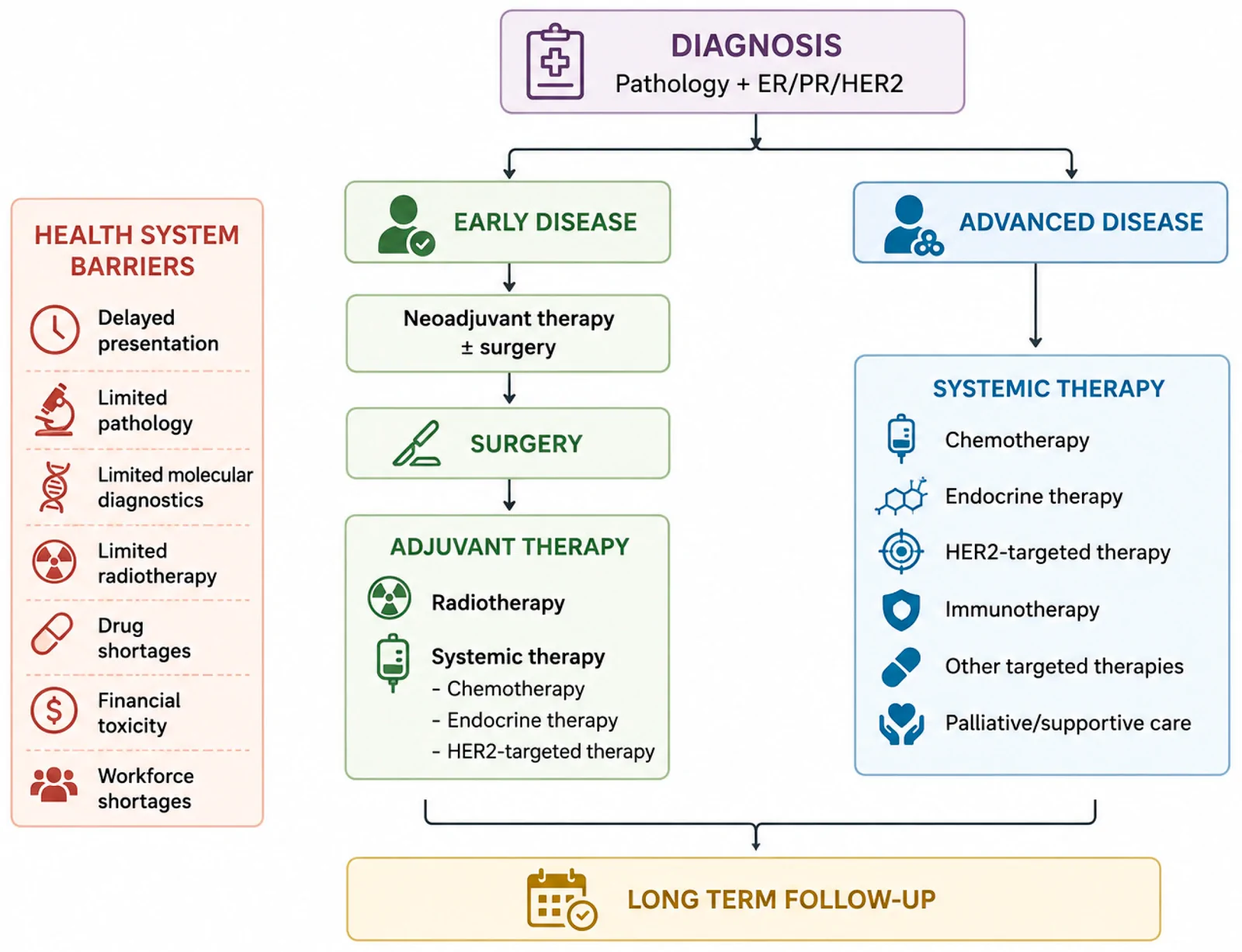
Breast cancer treatment pathway and major health-system barriers across Africa.

Although the fundamental principles of breast cancer management are consistent worldwide, their implementation throughout Africa is significantly influenced by healthcare infrastructure and resource availability. The tenets of breast cancer care, including multidisciplinary management involving surgeons, medical oncologists, radiation oncologists, pathologists, radiologists, oncology nurses, pharmacists, dietitians and supportive care specialists, remain the standard of care (Figure 2). However, many African countries face a severe shortage of specialised oncology personnel, resulting in substantial disparities in treatment access between African patients and those from the Global North, and between urban and rural patients. In very low-income countries, oncology services are concentrated within a few national referral centres, forcing patients to travel long distances and frequently contributing to treatment delays or abandonment [15,16].

Access to chemotherapeutics also varies considerably across the continent. While conventional cytotoxic chemotherapies, including anthracycline- and taxane-based regimens, may be available in many tertiary centres, interruptions in drug supply, inconsistent procurement systems, and limited availability of supportive medications frequently compromise treatment delivery. Access to newer, targeted therapies, including anti-HER2 agents, immune checkpoint inhibitors, poly(ADP-ribose) polymerase (PARP) inhibitors, and antibody–drug conjugates, is restricted in many African countries because of their high acquisition costs, limited reimbursement mechanisms for patients, and inadequate molecular diagnostic infrastructure required for patient selection [16,17].

The substantial financial burden associated with the high cost of medical care [18] represents another material barrier to optimal breast cancer care. In many, but not all, African healthcare systems, patients bear a substantial proportion of treatment costs through out-of-pocket payments. Apart from the medical diagnostic and treatment regimens, expenses for transportation, accommodation, and lost income often create catastrophic financial burdens for patients and their families. These economic challenges contribute to delayed treatment initiation, reduced adherence to prescribed therapies, and premature discontinuation of treatment, ultimately compromising clinical outcomes, as was shown in studies in Ethiopia and Nigeria [19,20].

Health-system bottlenecks remain an obstacle to timely and effective breast cancer treatment across many African countries [21]. Delays frequently occur throughout the care pathway, including delayed referrals from primary healthcare facilities to specialised oncology centres, long turnaround times for histopathological diagnosis and biomarker assessment, limited access to multidisciplinary team (MDT) discussions, and extended waiting lists for surgery and radiotherapy. Consequently, the interval between diagnosis and treatment initiation is often substantially prolonged, increasing the risk of disease progression, reducing treatment efficacy, and negatively affecting overall survival [19,21,22,23].

Despite these considerable challenges, breast cancer management in Africa is undergoing gradual transformation. Expansion of national cancer control programmes, increasing adoption of multidisciplinary care models, improved access to biosimilar medicines, regional oncology training initiatives, and growing participation in international clinical trials are beginning to improve treatment capacity in several countries. Furthermore, advances in molecular diagnostics, pharmacogenomics, precision oncology, immunotherapy, and locally relevant implementation research offer potential new opportunities to reduce disparities and improve outcomes. Sustained investment in healthcare infrastructure, workforce development, equitable access to essential medicines, and context-specific clinical research will be essential to ensure that advances in breast cancer therapeutics are translated into meaningful survival benefits for women across the African continent.

This review aims to examine recent advances in breast cancer diagnostics and therapeutics, while identifying evidence gaps and implementation challenges that must be addressed to advance context-appropriate breast cancer care in Africa.

## 2. Literature Search

A comprehensive literature review was conducted through three systematic search rounds targeting peer-reviewed scientific literature on breast cancer diagnostics and treatment in African countries published between January 2001 and August 2026. The search strategy was designed to capture the breadth of evidence across diagnostic technologies, treatment modalities, clinical outcomes, and barriers to care. This literature search was conducted using Web of Science and Google Scholar. The search was organised across five thematic frameworks: (1) breast cancer screening and early detection: clinical breast examination and mobile mammography services; (2) diagnostic imaging: mammography, ultrasonography, MRI, and emerging technologies; (3) tissue and molecular diagnostics: fine needle aspiration, core needle biopsy, histopathology, immunohistochemistry and liquid biopsy; (4) emerging and precision oncology: future directions in breast cancer diagnostics in Africa; (5) treatment modalities and barriers; surgery types, therapy regimens, barriers and challenges.

The search strings applied included combinations of breast cancer AND (“Sub-Saharan Africa” OR “Africa” OR “low-income countries” OR “resource-limited”). For diagnostic modalities: AND (“diagnosis” OR “screening” OR “detection”), this was further supplemented to include specific terms such as “MRI”, “liquid biopsy”, “artificial intelligence” OR “AI”, and “immunohistochemistry”. For treatment modalities: AND (“treatment” OR “therapy” OR “chemotherapy” OR “surgery” OR “radiotherapy” OR “hormone therapy” OR “targeted therapy” OR “immunotherapy”).

The included studies were all peer-reviewed and published in ISI journals between 2001 and 2026, except for three articles that are currently in preprint. Studies were excluded if they involved non-human model organisms (animal models, organoids, cell lines), male subjects, single case reports, and non-English language publications. Articles that reported purely in vitro studies were also excluded. Collected publications were checked for duplication via DOI matching and manual comparison between closely related studies.

Resulting deduplicated papers were ranked for relevance based on direct relevance to breast cancer diagnosis or treatment in Africa, quality of study design, specificity of African context, and contribution to understanding diagnostic technologies, treatment outcomes, or barriers.

Data extraction from each included study included study characteristics (country, design, sample size, and time period), diagnostic methods and performance metrics (sensitivity, specificity, and accuracy), treatment modalities across all relevant categories (surgery, chemotherapy, radiotherapy, hormone therapy, targeted therapies, and immunotherapy), and clinical outcomes including survival, response rates, recurrence, and toxicity. Additional domains encompassed barriers to care, health economic parameters, and care pathway indicators such as diagnostic intervals and treatment delays. Findings were synthesised narratively and organised thematically in accordance with the review structure. The result collection of articles covered all African regions, with the highest representation from South Africa, Nigeria, Ethiopia, Kenya, Ghana, and Uganda. Studies ranged from single-institution case series to multi-country prospective cohorts, systematic reviews, and implementation of research.

## 3. Current Diagnostic Modalities and Emerging Technologies

Despite substantial advances in breast cancer diagnostics globally, the African context presents unique challenges related to accessibility, affordability, workforce capacity, and validation in diverse populations. Emerging technologies offer promising opportunities to bridge these gaps, but implementation research and context-specific evidence remain limited.

### 3.1. Breast Cancer Screening and Early Detection

Breast cancer mortality increases in resource-limited settings where screening programmes are limited. Earlier diagnosis requires breast-health education for all women and a phased, resource-sensitive approach, beginning with training healthcare workers in clinical breast examination and progressing to opportunistic assessment and pilot screening programmes using clinical or self-examination [24]. Black and Richmond (2019) highlighted the extension of these principles in sub-Saharan Africa (SSA), given that most patients present with advanced-stage disease and face significant delays between symptom onset and clinical intervention [25].

#### 3.1.1. Clinical Breast Exam

In regions where population-based mammography programmes remain logistically and economically unfeasible, clinical breast examinations (CBEs) performed by trained healthcare providers serve as the primary cornerstone for improving survival rates [26]. In these settings, CBE is viewed not just as a screening tool, but as a vital mechanism for “clinical downstaging”, the process of detecting cancers earlier in symptomatic women to improve prognosis [25]. The primary promise of CBE lies in its accessibility and cost-effectiveness compared to high-tech alternatives. CBE combined with treatment was the most cost-effective intervention in Ghana, with a cost per disability-adjusted life year saved roughly 10 times lower than mammography screening [27].

CBE is a relatively simple technique that can be performed effectively by trained non-medical health workers, making it suitable for rural areas where specialists are scarce [25,28]. It consists of a systematic process of inspection and palpation of both breasts and axillae designed to identify palpable tumours at an earlier stage [29]. CBE is the first step in a screening triad that includes imaging (usually ultrasound or mammography), followed by diagnostic laboratory confirmation (such as Fine Needle Aspiration histology) [30].

CBE has been shown to contribute to a 17% to 47% reduction in advanced-stage cancer diagnosis [31]. This is critical in SSA, where up to 77% of cases are currently diagnosed at stage III or IV [26]. While mammography has higher sensitivity (77–95%), evidence suggests that a well-performed CBE (sensitivity 40–69%) can achieve a similar effect on mortality reduction in low-resource settings [31].

A cost-effectiveness analysis from South Africa evaluated twice-yearly CBE screening for asymptomatic women aged 40–69 years, as recommended by national policy [32]. While the study confirmed CBE as more affordable than mammography, questions remain about optimal screening intervals and age ranges for African populations. Training and quality assurance for CBE remain critical challenges, as performance is highly operator-dependent and requires ongoing skill maintenance [33,34]. Thus, despite its promise, several barriers hinder the effective use of CBE in Africa. For example, the scarcity of trained personnel and the lack of standardised training protocols often result in significant variability in diagnostic sensitivity and clinical downstaging outcomes [35]. Moreover, the successful scale-up of these efforts requires the integration of culturally appropriate awareness campaigns to mitigate persistent social stigma and misconceptions that prevent patients from seeking timely formal medical evaluation [36]. Low levels of awareness and deeply rooted misconceptions about breast cancer causes are prevalent. For example, in Ghana, while 60.4% of women had heard of CBE, only 12% had undergone targeted breast screening [37]. CBE alone is insufficient for early detection of small, non-palpable tumours, limiting its impact at the diagnosis stage. Systematic reviews of screening interventions in low- and middle-income countries emphasise that CBE should be viewed as a component of comprehensive breast health services rather than a standalone screening strategy [34].

#### 3.1.2. Mammography and Ultrasonography

The literature highlights a distinct dichotomy between the theoretical promise of imaging technologies and their practical application in Africa. While mammography and ultrasonography are foundational in high-income settings, their roles are being redefined to better serve the younger risk profile demographic and resource constraints of the African continent.

Mammography is recognised as the “gold standard” for early detection, with randomised controlled trials in high-income countries showing significant mortality reductions for women aged 50 and older [38]. However, its high cost and the requirement for specialised technical support often render it inaccessible for breast cancer screening in many African countries [39]. While imaging is essential for staging and management planning, population-based mammography is currently viewed as logistically and economically infeasible in most of SSA [40].

A major barrier against the use of mammography in Africa is the biological suitability of this technology in this demographic. The effectiveness is significantly impacted by the masking effect caused by dense breast tissue, which is particularly prevalent in younger women in Africa [25,41]. This is important since, in SSA, breast cancer patients are typically a decade or more younger than their Western counterparts [42,43,44]. Dense breast tissue contains high volumes of white-appearing glandular tissue that can mask a small malignancy due to insufficient contrast between the tumour and the healthy tissue [31]. Studies show that the sensitivity of mammography is significantly lower in extremely dense breast tissue when compared to fatty breast tissue [28]. As a result, the ambiguity of imaging in dense tissue often leads to false positives, triggering expensive and invasive follow-up procedures like biopsies that many patients cannot afford [28].

Breast ultrasonography, particularly Point-of-Care Ultrasonography (POCUS), is frequently recommended as a suitable adjunct or primary diagnostic tool for younger women in resource-limited settings [45,46,47]. Unlike mammography, ultrasound can distinguish solid tumours from dense fibroglandular tissue, offering up to 84% sensitivity for detecting palpable masses in these populations [39]. Evidence from Nigeria and Uganda supports the use of echography as a viable and cost-effective component of the triple assessment, which includes a clinical assessment followed by radiological imaging and histopathology [48].

A primary limitation of ultrasound is that it is highly operator-dependent [46,49]. There is a dearth of skilled radiologists reported in sub-Saharan Africa. Ultrasound equipment is more accessible than mammography equipment. For instance, Uganda reportedly has over 100 high-frequency ultrasound machines compared to just five mammography units. However, the lack of trained personnel to utilise them effectively remains a major hurdle [49,50].

#### 3.1.3. Mobile Screening Services

Mobile units have emerged as a strategic intervention to bypass the logistical barriers and transportation issues that often prevent rural populations from accessing centralised diagnostic facilities [42]. Programmes like PinkDrive in South Africa and broader integrated solutions like those provided by Radhiant Diagnostic Imaging South Africa (RDISA) represent a shift toward decentralised, community-based care in resource-limited settings [51].

PinkDrive is cited as a prime example of a successful public–private partnership (PPP) model, leveraging corporate social investment to fund mobile units that the government often cannot afford [52]. A major benefit of the PinkDrive model is its holistic approach. Rather than screening for breast cancer in isolation, mobile units offer integrated services for cervical and prostate cancer, alongside HIV/AIDS, TB, and non-communicable diseases like obesity, addressing a high prevalence of comorbidities in Africa [52,53]. While RDISA is less documented in peer-reviewed academic journals, its role aligns with the push for “decentralised diagnostic hubs” that use integrated radiology and AI-powered imaging to bridge the gap in fragmented healthcare systems [35,51].

A significant debate exists regarding the clinical quality of NGO-led mobile units. In 2013, Bateman [54] from the Breast Interest Group of South Africa criticised PinkDrive’s intervention, citing concerns over poor imaging quality, inaccurate interpretation, and a failure to operate under rigorous scientific scrutiny. Some experts suggest that screening via mobile units is ineffective if it is not coupled with a functioning system for follow-up and treatment [25,55]. Evidence from Ghana suggests that identifying patients through community screening often still results in late-stage treatment because the subsequent diagnostic work-up (biopsies and pathology) is not integrated or affordable [42].

#### 3.1.4. Magnetic Resonance Imaging

Breast magnetic resonance imaging (MRI) is established as one of the most sensitive imaging modalities for breast cancer detection, even though its widespread adoption is limited by cost and availability [56]. Reported sensitivity in international multicentre studies of high-risk women ranges from 77% to 100%, exceeding other imaging modalities [56,57]. However, specificity is variable, ranging from 81% to 99% in international studies [57]. Other meta-analytic data show a pooled sensitivity of 90% (95% CI: 0.88–0.92) and specificity of 72% (95% CI: 0.67–0.77) [58]. A direct African prospective MRI study from Uganda demonstrated sensitivity of 70%, specificity of 100%, positive predictive value of 100%, and negative predictive value of 73.9% using abbreviated dynamic contrast-enhanced MRI protocols, indicating an exceptional capability to differentiate between benign and malignant masses [59].

MRI is described as a logistically challenging modality in African settings, and the literature frames it predominantly as a staging and management tool rather than a screening tool, much like in the rest of the world. However, even for staging and management, MRI technology is not widely used in Africa. In 2017, the WHO African Region had only 0.7 MRI scanners per million people (pmp), the lowest density among all six WHO-defined regions. This compares to 5.4 MRI scanners pmp in the Asia Western Pacific Region and 11.6 in the Eurasian Region [60]. In 2016, only 84 MRI units were serving West Africa’s population of more than 370 million, with more than two-thirds of these located in Nigeria [60]. MRI is rarely available at no cost to the patient in African healthcare systems, making out-of-pocket costs prohibitive for most patients [61]. Even where an MRI machine is procured, the system still suffers from high image acquisition costs, limited maintenance infrastructure, and a lack of the technical expertise required to operate and maintain the equipment [60].

Notwithstanding these difficulties, MRI may be uniquely suited to the African presentation phenotype. Its suitability stems from MRI’s lack of sensitivity loss in dense breast tissue, unlike mammography, making it potentially advantageous [62]. Recent feasibility studies show that ultra-low field (ULF) unilateral breast MRI (performed on 11 healthy women without contrast) can reliably delineate fibroglandular tissue from adipose tissue and detect benign masses [63]. However, the higher MRI PPV than that of mammography does not eliminate the need for subsequent diagnostic confirmation via biopsy [62].

### 3.2. Tissue and Molecular Diagnostics

Tissue and molecular diagnostics remain indispensable to effective clinical management of breast cancer. A central principle underpinning breast cancer care policy is that confirmation of breast cancer diagnosis, regardless of what imaging modality is used, ultimately requires adequate anatomic pathology laboratories that allow for quality processing of the sampled tissues [64]. The Breast Health Global Initiative emphasises that the triple test (combining clinical breast examination, diagnostic imaging, and tissue pathology) has proven reliable with high sensitivity and specificity, even in resource-limited countries such as sub-Saharan Africa [64,65].

#### 3.2.1. Fine Needle Aspiration and Core Needle Biopsy

A study by Ziegenhorn, Frie [66] positions FNA as the dominant routine diagnostic technique in African pathology centres, performed daily in 50% of centres profiled, due to its low cost and minimal equipment requirements. However, few African studies directly compare FNA and CNB diagnostic accuracy, sensitivity, and treatment impact in the same patient cohort, leaving clinicians to make technique choices without local evidence [42,66]. While CNB is preferred worldwide, research on the development of affordable CNB in primary African settings is sparse. It is often noted that core needles remain costly and ultrasound guidance for CNB is often unavailable, but little actionable implementation evidence is offered [66].

Furthermore, the biopsy-related literature remains heavily concentrated in West Africa, South Africa, Kenya, and Namibia; Central and parts of North Africa are virtually unrepresented [25,67]. A consequence of this is that few African studies link biopsy technique to definitive survival outcomes. For example, the Toma, O’Neil [68] study is one of the largest LMIC histopathology audits but does not correlate the biopsy pathway with disease-specific survival. A final crucial gap is that patient preferences, cultural sensitivities (e.g., breast conservation concerns), and the acceptability of repeat biopsies are extremely understudied in African populations [36,69].

#### 3.2.2. Histopathology and Immunohistochemistry

Histopathology and immunohistochemistry (IHC) are the foundational molecular diagnostic pillars supporting breast cancer management in Africa. IHC can identify the disease, classify its molecular subtype, and direct targeted therapy.

In breast cancer, the standard IHC panel includes oestrogen receptor (ER), progesterone receptor (PR), human epidermal growth factor receptor 2 (HER-2) and Ki-67 proliferation index [70].

Histopathology and IHC together fulfil three indispensable functions in breast cancer management. The first is the confirmation of diagnoses. While imaging identifies a lesion, only pathology can definitively confirm whether cells are malignant. Secondly, prognostic stratification can be delineated, since histologic grade, lymph node involvement, lymphovascular invasion, and Ki-67 proliferation index together provide prognostic information that guides the intensity of adjuvant chemotherapy and overall survival estimates [68]. Finally, histopathology and IHC are crucial for directing treatment. Since breast cancer is a biologically heterogeneous group of malignancies, treatment depends on subtype [67]. Without IHC, clinicians cannot rationally allocate these very different treatment pathways, a problem compounded in LMICs where drug access itself is constrained.

A finding from earlier research showed that African breast cancer patients often were offered hormonal therapy even though their hormone receptor status was unknown due to a lack of IHC services [71]. Without accurate IHC, hormonal therapy can be prescribed to receptor-negative patients (ineffective) or withheld from receptor-positive patients (missing an effective treatment). Given the variability in IHC quality, up to 20% of analyses result in false positives and negatives even in developed countries; the African rate is likely higher [71]. Consequently, the apparent biology of African breast cancers, which is widely cited as predominantly triple-negative, may be significantly confounded by inadequate tissue processing [42,72].

A survey conducted by Ziegenhorn, Frie [66] of 20 SSA pathology centres revealed that 14 (70%) reported temporary power outages, severely affecting tissue processing, fixation, and staining [66]. The survey further reported that formalin-fixed paraffin-embedded (FFPE) blocks were prepared with pure paraffin in 14 (74%) of the participating centres, but with paraffin and additives in 3 (16%) and even with candle wax in 2 (11%) of the centres [66]. The use of candle wax, a non-standard paraffin substitute, suggests fundamental reagent-supply unreliability.

Importantly, evidence suggests that tissue handling may influence antigenicity. A Nigerian study using surgically excised tumours showed ER-negative rates of approximately 80%, whereas a study using CNBs (which fix faster) showed ER-negative rates of approximately 27% in the same region [42]. The difference reflects pre-analytical variables that caused delays before fixation due to larger surgical specimens, which leads to reduced ER antigenicity, while smaller CNB cores are rapidly fixed and, as a result, preserve antigenicity [42,72].

Rwanda conducted a cost and comparative analysis and between IHC and the novel Xpert^®^ STRAT4 Assay, a GeneXpert^®^-based mRNA expression assay [73]. STRAT4 quantifies mRNAs for ESR1, PGR, ERBB2, and MKi67. The study showed the assay had a high concordance with IHC and fluorescence in situ hybridisation (overall percentage agreements of 98%, 90%, and 93% for ESR1, PGR, and ERBB2, respectively). The GeneXpert^®^ platform, already deployed across Africa for tuberculosis diagnosis, therefore offers potential for decentralised breast cancer molecular diagnostics.

#### 3.2.3. Liquid Biopsy

Liquid biopsy is a non-invasive diagnostic technique that detects and analyses tumour-derived material shed into bodily fluids, most commonly peripheral blood [74]. Unlike surgical or needle biopsy, which requires removing a piece of tissue, liquid biopsy exploits the fact that malignancies shed detectable material into circulation. These include circulating tumour cells (CTCs), which are intact tumour cells that have detached from the primary tumour and entered the bloodstream. CTCs are isolated from the blood using surface markers and quantified (e.g., CD14, CD133, VEGFR2) or analysed by molecular methods [74]. Cell-free genetic material, including cell-free DNA (cfDNA) and circulating tumour DNA (ctDNA), can also be isolated from blood. These are fragmented pieces of DNA released by tumour cells, which can be analysed through nucleic acid amplification or next-generation sequencing to identify tumour-specific mutations [74,75]. Furthermore, liquid biopsies can detect circulating RNAs, such as mRNAs or microRNAs, extracellular vesicles and exosomes and tumour-associated proteins.

Liquid biopsies could address several structural problems unique to the African continent. For example, because surgical biopsy is invasive and constrained in low-resource settings, “the use of liquid biopsy for non-invasive cancer diagnosis can have pivotal importance for the African continent where access to medical infrastructures is limited, as it eliminates the need for surgical biopsies” [74]. Additionally, this technique offers the potential for real-time monitoring since it enables observation of treatment response, relapse, and metastatic evolution, features especially valuable in SSA where post-treatment surveillance pathways are weak [76]. Finally, a simple peripheral-blood draw is far more acceptable and logistically simpler than a surgical biopsy, aligning with the SSA reality that patients may travel hundreds of kilometres for care [36].

While the promise is genuine, the literature repeatedly emphasises that the prospects, barriers, and challenges have not been fully addressed for African deployment [76]. A significant limitation for ctDNA mutational analysis in Africa is the genetic diversity of the African population and variability in gene expression among different populations, because it is unclear whether the cancer-related mutations and gene expression ranges validated in European-ancestry cohorts apply to African patients [77]. Moreover, high costs of high-coverage sequencing are one of three named major obstacles (alongside infrastructure and skills) facing SSA adoption of liquid biopsy technology. Reagents for ctDNA isolation, library preparation, and NGS are expensive. African studies such as Ahuno, Doebley [75] had to leverage international collaborations with Mount Sinai, Fred Hutchinson, and the NCI to perform liquid biopsy studies, indicating that independently resourced African-led liquid-biopsy implementation studies remain difficult to execute.

An important drawback of liquid biopsies is the limited sensitivity at low tumour burdens. Globally, ctDNA detection sensitivity is lower in early-stage disease because of low tumour shedding. In SSA, where many patients present with advanced-stage disease (e.g., Uganda reports 87% advanced presentation with 5% annual incidence increase), this lower threshold may be less important due to the late-stage presentation [78]. CTC counts in early breast cancer may be below the threshold of detection by CellSearch or flow cytometry platforms, limiting utility for screening [74].

Liquid biopsy offers unique structural advantages for the African continent. However, its benefits will only be fully realised through African-specific validation studies, multidisciplinary workforce development, integration with existing hub-and-spoke pathology models, and parallel investment in conventional IHC infrastructure that remains the African diagnostic bottleneck today [36,66,71,79].

### 3.3. AI Technologies and Future Directions

#### AI-Assisted Diagnostics

The integration of artificial intelligence (AI) into diagnostic workflows has the potential to mitigate the chronic shortage of sub-specialised pathologists and radiologists across the African continent. By leveraging telepathology and whole-slide imaging, these digital tools enable remote assessment and collaborative reporting, effectively extending the reach of limited expert human resources [72]. By providing scalable, cost-effective computational support, AI-driven solutions may effectively redress prevailing healthcare disparities, even if their current clinical sensitivity and specificity require further refinement [80].

AI integration is perhaps the most actively researched frontier in African imaging. A South African prospective study by Malherbe [39] at Daspoort PoliClinic in Gauteng screened 1617 women aged 25 to 85 using both CBE and Breast AI. It was found that Breast AI, the SAPHRA-approved AI-enhanced diagnostic tool, demonstrated clinical utility as an adjunct to clinical breast examination and showed promise for clinical downstaging and reducing unnecessary surgical referrals [39]. The use of breast ultrasound alongside an AI algorithm has been shown to increase accuracy rates by over 12% for breast cancer detection and is considered a viable and cost-effective method for screening in resource-constrained public healthcare clinics in southern Africa [39]. Notably, the South African Breast Cancer Commission authors explicitly advocate for AI-powered imaging integrated into decentralised diagnostic hubs to bridge fragmented healthcare systems in LMICs [51].

A study conducted by Pace, Dusengimana [81] at The Breast Ultrasound Initiative at Butaro District Hospital in Rwanda trained four general practitioners and five nurses in diagnostic breast ultrasound with AI-assisted interpretation support from Boston-based radiologists. The study demonstrated that diagnostic breast ultrasound by general practitioners and nurses can be a useful tool in the evaluation of breast lesions, including palpable masses, at a rural cancer facility in Rwanda. Early findings suggest that it has allowed avoidance of biopsy for 42% of patients with breast masses noted on ultrasound [72]. While this is not strictly an AI study, it established the foundational workflow on which AI augmentation builds.

Multiple studies from Burkina Faso have developed AI platforms for breast cancer detection from histopathological images [82,83]. These systems utilise convolutional neural networks and deep learning algorithms to automate pathology image analysis, addressing challenges of low image quality and limited pathologist availability. While specific performance metrics were not consistently reported, the studies demonstrate the potential feasibility of AI implementation in West African settings.

Gueye, Gueye [84] developed an AI system for histopathological diagnosis and grading of breast cancer in Ethiopia [84]. The deep learning-based classification system achieved 96.75% accuracy for binary classification, 96.7% for benign subtype classification, 95.78% for malignant subtype classification, and 93.86% for grade identification [84]. These high accuracy rates suggest potential for AI to augment limited pathology capacity.

MAMMOAI is a Kenyan-developed AI framework explicitly designed for breast cancer detection, with applicability in Africa. The technology uses a hybrid ResNet50-Transformer backbone integrating convolutional neural networks with transformer layers for simultaneous risk assessment, cancer detection, staging, risk factor analysis, and differential diagnosis [85]. The model was trained using the KAU-BCMD dataset from Saudi Arabia, supplemented with preliminary Kenyan data because larger African mammography datasets were unavailable. The model achieved 98% accuracy on BI-RADS 1 (majority class); however, it achieved only macro-averaged F1-scores of 0.61–0.62 across all branches, indicating challenges with minority classes [85]. A comprehensive table of AI technology advances in Africa can be found in Table 1.

There are several benefits of AI-assisted technologies specific to African clinical realities. Firstly, AI tools can fill critical gaps where there are shortages of trained personnel. Rwanda’s BUS initiative has shown that nurses and GPs can produce diagnostic-quality AI-assisted scans with mentorship from distant radiologists [81,86]. Furthermore, AI integration with portable ultrasound or low-cost platforms yields cost-effective screening for resource-constrained public healthcare clinics [39,89]. In addition to cost-effectiveness, AI can reduce diagnostic workflow time through automated triage, allowing fewer specialists to manage higher volumes.

There remain, however, several barriers to overcome with the implementation of AI for diagnosis throughout Africa. The lack of high-quality, expert-labelled breast ultrasound datasets poses a major barrier to applying machine learning in clinical settings. To date, there have been no known efforts to develop machine learning models for computer-aided screening, diagnosis, or risk assessment of breast cancer specifically in Sub-Saharan women [49]. This remains the single most cited limitation across the literature: every African AI breast cancer project must rely on non-African datasets for initial training.

Another barrier pertains to infrastructural prerequisites. AI platforms require digital scanners, internet connectivity, computer hardware/server capacity for inference, and stable electricity, all of which are unreliable in many SSA contexts [88].

AI can plausibly transform African breast cancer screening by democratising access to diagnostic-grade imaging, but its clinical deployment in the African context still requires substantially more validation, cost-effectiveness data, ethical regulation, and integration with the existing triple-test pathway. Until those gaps are closed, AI-assisted technologies should be complementary rather than transformative in African breast cancer diagnostics.

## 4. Current Therapeutic Management and Emerging Treatments

### 4.1. Surgical Management of Breast Cancer in Africa

Surgery remains the cornerstone of curative treatment for early-stage breast cancer and is an essential component of multidisciplinary breast cancer management. The primary objectives of surgery are complete tumour excision, accurate pathological staging, and local disease control while preserving quality of life whenever possible [90,91]. Surgical management has evolved considerably over the past three decades, shifting from radical procedures towards personalised, less invasive approaches. However, despite these global advances, surgical practice across Africa continues to be shaped by resource limitations, delayed diagnosis, and disparities in access to specialised breast care services [91,92].

Modified radical mastectomy remains the most frequently performed surgical procedure for breast cancer in many African countries. Unlike breast-conserving surgery, mastectomy removes the entire breast and generally does not routinely require postoperative radiotherapy for every patient, making it a more practical option in resource-limited settings [90,93]. Advanced-stage presentation, large tumour size, multifocal disease, and delayed diagnosis often necessitate complete breast removal to achieve local disease control. Furthermore, limited access to neoadjuvant systemic therapy may reduce opportunities to downstage tumours sufficiently for breast conservation [94,95].

Breast-conserving surgery (BCS), also referred to as lumpectomy or wide local excision, involves excision of the tumour with an adequate margin of surrounding healthy tissue while preserving most of the breast. Multiple randomised clinical trials have demonstrated that BCS followed by adjuvant radiotherapy provides overall survival equivalent to mastectomy in appropriately selected patients with early-stage breast cancer [96,97]. Consequently, BCS has become the preferred surgical approach for many women with Stage I and II disease in high-income countries.

Despite these advances, the implementation of BCS in Africa remains limited. A major challenge is that many women continue to present with large, locally advanced tumours (Stage III or IV), making breast conservation unsuitable at diagnosis. Furthermore, for many women, the success of BCS depends on timely access to postoperative radiotherapy to minimise local recurrence. However, radiotherapy services remain severely constrained across much of SSA, with limited treatment centres concentrated in urban areas, prolonged waiting times, equipment shortages, frequent machine breakdowns, and significant travel distances for many patients. Together, these health-system barriers substantially reduce the feasibility and uptake of breast-conserving approaches across the region [95,98].

Axillary lymph node dissection (ALND) has historically been the standard approach for regional nodal staging; however, it is associated with significant postoperative morbidity, including lymphoedema, shoulder dysfunction, chronic pain, and sensory impairment [99]. Globally, sentinel lymph node biopsy (SLNB) has become the standard of care for patients with clinically node-negative breast cancer, offering equivalent staging accuracy while substantially reducing surgical morbidity compared with ALND. However, adoption of SLNB across Africa remains uneven because its implementation requires specialised nuclear medicine facilities, radiotracers or alternative lymphatic mapping agents, experienced multidisciplinary teams, and reliable pathology services, all of which are unavailable in many healthcare settings [100]. Consequently, ALND continues to be widely performed in numerous African centres, particularly for patients presenting with clinically node-positive disease or where SLNB technology is inaccessible [100].

Encouragingly, recent real-world evidence from North Africa demonstrates that SLNB can be safely and effectively implemented in resource-constrained settings. In a retrospective study, Belkhodja et al. [101] reported that SLNB provided reliable axillary staging with acceptable oncological outcomes while reducing the need for routine ALND and its associated morbidity. These findings suggest that contemporary, less invasive axillary staging is achievable in selected African centres when appropriate infrastructure, pathology support, and surgical expertise are available.

Breast reconstruction has become an integral component of comprehensive breast cancer care in many high-income countries. Reconstruction may be performed immediately following mastectomy or delayed until completion of adjuvant therapy, using either implant-based or autologous tissue techniques [102].

Despite these benefits, reconstructive surgery remains inaccessible for most women across Africa and is often regarded as a luxury rather than a standard component of breast cancer treatment. Improving access to reconstructive services represents an important opportunity to enhance survivorship care and address the long-term psychosocial consequences of breast cancer treatment [95]. Immediate reconstruction remains uncommon because many women present with locally advanced disease requiring adjuvant therapy, which may influence the timing and feasibility of reconstruction.

### 4.2. Radiotherapy

Radiotherapy is an integral component of multidisciplinary breast cancer management and plays a critical role in improving local tumour control, reducing recurrence, and enhancing long-term survival. Approximately 50–60% of patients with breast cancer require radiotherapy during their disease, either with curative or palliative intent [103]. Advances in treatment planning, image-guided radiotherapy, and modern radiation delivery techniques have substantially improved treatment precision while minimising radiation exposure to surrounding normal tissues. Despite these technological advances, access to radiotherapy remains one of the greatest barriers to equitable breast cancer care across Africa [103].

Adjuvant radiotherapy following surgical resection represents the most common indication for radiotherapy in breast cancer management. After BCS, whole-breast irradiation is the standard of care because it significantly reduces local recurrence while achieving overall survival comparable to mastectomy [104]. Postmastectomy radiotherapy is recommended for patients with high-risk pathological features, including large primary tumours, positive surgical margins, and extensive axillary lymph node involvement. In appropriately selected patients, postoperative radiotherapy improves locoregional control and contributes to better disease-free and overall survival [105].

Neoadjuvant radiotherapy involves delivering targeted radiation prior to surgical intervention to downstage bulky or locally advanced tumours, thereby converting previously inoperable disease into resectable candidate cases and improving surgical margin control. The routine use of neoadjuvant radiotherapy in breast cancer remains limited. It is generally reserved for carefully selected patients with unresectable or locally advanced disease when surgery is not initially feasible or within clinical trial settings evaluating novel treatment strategies [103].

The requirement for adjuvant radiotherapy influences surgical decision-making. South African breast cancer survivors reported that the need for post-operative radiation factored into their treatment decisions, with many opting to undergo a mastectomy due to radiotherapy access concerns [106]. This creates a paradox where breast-conserving surgeries, which improve both cosmetic outcomes and quality of life, are not a feasible option.

Limited radiotherapy infrastructure and workforce capacity remain major barriers to equitable cancer care. Linear accelerators (LINACs), the global standard for external beam radiotherapy, remain scarce in many African countries, and existing facilities often operate beyond capacity. A recent multi-country survey involving 25 African countries highlighted substantial disparities in radiotherapy resources, identifying only 43 LINACs and 18 cobalt-60 teletherapy units among participating countries. Southern and North Africa accounted for the greatest availability of LINACs, whereas East Africa relied more heavily on cobalt-60 technology, reflecting persistent regional inequities in access to modern radiotherapy services [107]. The survey also revealed a relatively young radiotherapy workforce, with a large proportion of practitioners having fewer than four years of professional experience, underscoring the urgent need for continued workforce development and specialist training to meet the continent’s increasing cancer burden [107].

In addition to limited infrastructure, operational challenges continue to compromise the delivery of radiotherapy services. Frequent equipment breakdowns, inadequate maintenance programmes, shortages of replacement parts, and unreliable electricity supplies contribute to interruptions in treatment. Furthermore, radiotherapy centres are predominantly located in major urban areas, requiring many patients to travel long distances for daily treatment over several weeks. These logistical and financial barriers frequently result in delayed treatment initiation, reduced adherence, or treatment abandonment. In the multi-country survey by Mallum, Sibanda [107], the average waiting time ranged from approximately four weeks in South Africa to more than ten weeks in Cameroon, illustrating the substantial disparities in timely access to radiotherapy services [107]. Such delays are particularly detrimental following BCS, where postponement of adjuvant radiotherapy has been associated with an increased risk of local recurrence and poorer clinical outcomes [103].

Despite these challenges, several developments provide opportunities to strengthen radiotherapy services across Africa. The increasing adoption of evidence-based hypofractionated radiotherapy can shorten treatment duration, improve patient convenience, reduce healthcare costs, and increase machine capacity without compromising oncological outcomes. At the same time, ongoing investment in radiotherapy infrastructure, workforce training, equipment maintenance, and the establishment of regional cancer centres will be essential to address existing service gaps. Emerging technologies, including artificial intelligence-assisted treatment planning, image-guided radiotherapy, and adaptive radiotherapy, also offer opportunities to improve treatment precision and operational efficiency. Together, these innovations have the potential to expand access, optimise resource utilisation, and support more equitable delivery of high-quality breast cancer care across Africa [108].

### 4.3. Systemic Therapy

Systemic therapy forms the backbone of modern breast cancer management and has substantially improved survival across all molecular subtypes. Contemporary treatment strategies include chemotherapy, endocrine therapy, targeted therapies, immunotherapy, and more recently, antibody–drug conjugates and precision medicine approaches [109].

In Africa, however, access to systemic therapies remains highly heterogeneous. While conventional chemotherapy is widely available in many tertiary hospitals, access to targeted therapies and novel biologic agents remains limited because of high costs and restricted laboratory capacity. Consequently, treatment decisions are frequently influenced not only by tumour biology but also by healthcare infrastructure and drug availability [110].

#### 4.3.1. Chemotherapy

Chemotherapy remains the most widely available systemic treatment for breast cancer across Africa and continues to be the cornerstone of treatment for triple-negative breast cancer (TNBC) [111], locally advanced disease, and many patients with high-risk early-stage breast cancer. Anthracycline- and taxane-based regimens remain the global standard of care and constitute the backbone of treatment protocols in most African oncology centres [112,113].

Adjuvant chemotherapy is administered following definitive surgery to eradicate residual microscopic disease and reduce the risk of recurrence. Treatment recommendations are based on tumour size, lymph node involvement, histological grade, molecular subtype, and increasingly, genomic risk assessment [113]. Anthracycline- and taxane-containing regimens remain the standard adjuvant treatment for patients with high-risk early breast cancer [113].

In many African countries, relatively few patients are eligible for primary surgery because of advanced-stage presentation. Evidence from Ghana indicates that women receiving adjuvant chemotherapy generally presented with earlier-stage disease and experienced more favourable survival outcomes than those requiring neoadjuvant treatment, underscoring the prognostic advantage of early diagnosis rather than an intrinsic benefit of treatment sequencing [114]. Nevertheless, timely initiation of adjuvant chemotherapy remains challenging because of delays in pathology reporting, limited oncology capacity, financial constraints, interruptions in drug availability, and prolonged intervals between surgery and systemic treatment, all of which may reduce treatment effectiveness.

Neoadjuvant chemotherapy (NACT) is administered before surgery to reduce tumour burden, increase operability, and facilitate breast-conserving surgery in selected patients. It has become the preferred approach for patients with locally advanced breast cancer, inflammatory breast cancer, HER2-positive disease, and TNBC [115,116].

In Africa, neoadjuvant chemotherapy has clinical importance because a substantial proportion of women present with locally advanced (Stage III) disease, precluding immediate surgery. A retrospective study from Ghana demonstrated that NACT enabled tumour downstaging and subsequent surgical treatment in many patients, although survival remained largely determined by disease stage at presentation and successful completion of multimodal therapy [114]. These findings highlight that the effectiveness of NACT in African settings depends not only on chemotherapy delivery but also on timely access to surgery, radiotherapy, and supportive care following systemic treatment [114].

For patients with metastatic breast cancer, chemotherapy aims to prolong survival, control tumour progression, alleviate symptoms, and maintain quality of life. Sequential single-agent chemotherapy is generally preferred to minimise toxicity while preserving quality of life, although combination regimens may be indicated in patients with rapidly progressive or life-threatening disease [117].

Across Africa, management of metastatic disease is complicated by late presentation, limited access to targeted therapies, inadequate palliative care services, and financial constraints. Consequently, conventional cytotoxic chemotherapy often remains the principal systemic treatment option for advanced disease [17,118].

Anthracycline-based regimens, including doxorubicin and epirubicin, are widely available in most tertiary oncology centres. Taxanes, including paclitaxel and docetaxel, are increasingly incorporated into standard treatment protocols but remain inconsistently available in lower-resource settings. Platinum agents, particularly carboplatin, have assumed an increasingly important role in the treatment of TNBC but remain unavailable or inconsistently supplied in several healthcare systems. Capecitabine access varies considerably between countries [110].

Delivering chemotherapy safely and effectively remains dependent on healthcare infrastructure. Beyond drug procurement, chemotherapy requires specialised infusion facilities and staff. A recent economic evaluation conducted at a South African public tertiary hospital demonstrated that these service components contribute substantially to the overall provider cost of chemotherapy, highlighting that strengthening oncology infrastructure is as important as ensuring medicine availability [119].

Beyond delayed treatment initiation, ongoing therapeutic adherence is severely hindered by systemic supply-chain disruptions and acute financial constraints. One Nigerian study found that of patients who were eligible for chemotherapy, only 48% completed treatment [120]. Consequently, chemotherapy delivery across Africa continues to be constrained by intermittent medicine shortages, inadequate supportive care services, limited toxicity monitoring, shortages of specialised oncology personnel, and both patient- and health system-level financial pressures. These barriers contribute to treatment delays, dose modifications, incomplete chemotherapy courses, and disparities in treatment outcomes across the continent [119].

#### 4.3.2. Endocrine Therapy

Endocrine therapy is the cornerstone of systemic treatment for hormone receptor-positive (HR+) breast cancer, which accounts for approximately 70% of all breast cancer cases worldwide [121].

Tamoxifen continues to be the most widely prescribed endocrine agent because of its affordability, broad availability, and inclusion in many national essential medicines lists [122]. Although aromatase inhibitors, including letrozole, anastrozole, and exemestane, are increasingly incorporated into national treatment guidelines, their availability remains inconsistent in many resource-limited settings [122]. Likewise, access to ovarian function suppression using gonadotropin-releasing hormone agonists is frequently restricted by cost and limited availability, reducing opportunities to implement contemporary risk-adapted endocrine treatment strategies [123].

Despite the relative availability of endocrine therapy, ensuring long-term treatment adherence remains a major challenge across many African healthcare systems. Endocrine therapy is typically prescribed for five to ten years, and sustained adherence is essential to achieve the reductions in recurrence and mortality demonstrated in clinical trials. A qualitative study from Botswana found that access to and utilisation of endocrine therapy were influenced by multiple patient-, provider-, and health system-level factors [123]. Importantly, treatment discontinuation was more frequently associated with limitations in healthcare access and medicine availability than with medication-related adverse effects [123]. However, a care quality evaluation performed in South Africa found that 72% of HR+ patients received endocrine therapy within 365 days, approaching but not meeting international quality benchmarks [124].

Collectively, these findings demonstrate that improving endocrine therapy outcomes in Africa requires more than ensuring the availability of essential medicines.

#### 4.3.3. HER2-Targeted Therapy

Approximately 15–20% of breast cancers overexpress human epidermal growth factor receptor-2 (HER2) [7], a molecular subtype historically associated with aggressive disease and poor prognosis [125]. The introduction of HER2-targeted therapies has dramatically transformed clinical outcomes, converting HER2-positive breast cancer into one of the most treatable molecular subtypes [126].

Trastuzumab remains the standard first-line anti-HER2 therapy in both early-stage and metastatic disease and is commonly administered in combination with chemotherapy. Additional HER2-targeted agents, including pertuzumab, trastuzumab emtansine (T-DM1), and trastuzumab deruxtecan (T-DXd), have further improved survival in patients with high-risk or metastatic disease [125].

Despite these advances, access to HER2-targeted therapies remains one of the greatest therapeutic inequalities across Africa [16]. High acquisition costs limit national reimbursement programmes, inadequate healthcare funding, and inconsistent availability of HER2 testing substantially restrict patient access. In many low-resource settings, patients with HER2-positive disease continue to receive chemotherapy alone because targeted therapy is either unavailable or unaffordable [127].

Crucially, realising the clinical efficacy of anti-HER2 regimens relies not merely on procurement, but on scaling accessible diagnostic testing to accurately subtype the estimated 15–25% of patients whose HER2 status currently goes unprofiled. Without routine biomarker confirmation, high-cost targeted therapeutics risk being either underutilised or inappropriately allocated.

#### 4.3.4. PARP Inhibitors

Poly (ADP-ribose) polymerase (PARP) inhibitors represent an important example of precision oncology for patients carrying germline BRCA1 or BRCA2 pathogenic variants. By exploiting defects in homologous recombination DNA repair, PARP inhibitors selectively induce tumour cell death while largely sparing normal cells [128].

Olaparib and talazoparib have demonstrated significant improvements in progression-free survival among patients with HER2-negative metastatic breast cancer harbouring germline BRCA mutations [129].

Notwithstanding the relatively high prevalence of hereditary breast cancer in some African populations, access to PARP inhibitors remains extremely limited. The major barriers include restricted access to BRCA genetic testing, limited genetic counselling services, high treatment costs, inadequate reimbursement mechanisms, and the underdeveloped genomic infrastructure required to identify eligible patients. Expanding molecular diagnostic capacity, hereditary cancer services, and equitable access to targeted therapies will be essential for improving outcomes in patients with BRCA-associated breast cancer [130].

#### 4.3.5. Antibody–Drug Conjugates

Antibody–drug conjugates (ADCs) have emerged as a major therapeutic advance in breast cancer management, representing one of the most rapidly evolving areas of precision oncology. These agents combine tumour-specific targeting with monoclonal antibodies with highly potent cytotoxic payloads, enabling targeted drug delivery to cancer cells while minimising systemic toxicity. This targeted approach has expanded treatment options for patients with advanced breast cancer who have progressed on conventional systemic therapies [131].

In spite of these advances, access to ADCs across Africa remains extremely limited. Their widespread implementation is constrained by high acquisition costs, limited reimbursement mechanisms, restricted access to HER2 and other companion diagnostic testing, and the specialised oncology infrastructure required for their safe administration and monitoring.

### 4.4. Pharmacogenomics and Drug Toxicity

Pharmacogenomics, which examines how genetic variation affects drug response, has particular relevance for African populations given their high genetic diversity [132]. Genetic variation affecting drug metabolism and response is increasingly recognised as an important factor in treatment outcomes, with African populations exhibiting distinct pharmacogenetic profiles that influence chemotherapy toxicity and efficacy.

#### 4.4.1. Genetic Variation in Drug Metabolism

Pharmacogenetics reviews emphasise that genetic variability in drug metabolism may cause reduced patient adherence due to toxicity or sub-therapeutic doses. Variant alleles affecting drug metabolism are specific to or occur at higher frequencies in black African populations [133].

Botswana’s pharmacogenetics study documented preliminary data showing non-negligible rates of subjects with defective drug metabolism [134,135]. The study emphasised that genomic modulation of drug response, specifically pharmacogenetic profiles affecting therapeutic outcomes, compliance, and toxicity, is a significant problem requiring attention. High mortality despite relatively low incidence, younger median age, advanced-stage presentation, and high risk of treatment failure characterise breast cancer in Botswana.

Ethiopia’s study provided concrete evidence of pharmacogenetic impact on treatment outcomes [136]. CYP2J2*7 genotype predicts risk of chemotherapy-induced hematologic toxicity and reduced relative dose intensity in Ethiopian breast cancer patients. The CYP2J2*7 allele and low baseline blood counts increase risk for chemotherapy-induced hematologic toxicities and reduced relative dose intensity. This variant occurs at higher frequency in black African populations.

Tamoxifen requires metabolic activation by the cytochrome P450 enzyme CYP2D6 to form its active metabolite, endoxifen, and genetic variation in CYP2D6 can influence treatment efficacy and toxicity. Recent pharmacogenomic studies have demonstrated substantial CYP2D6 genetic diversity across sub-Saharan African populations, with clinically relevant variants and predicted metaboliser phenotypes differing from those commonly observed in European populations. These findings suggest that pharmacogenomic algorithms developed using predominantly non-African populations may not accurately predict tamoxifen metabolism in African patients, highlighting the need for population-specific pharmacogenomic evidence [137]. A subsequent review similarly concluded that pharmacogenetic data remain limited for women of African ancestry despite the continent’s extensive genetic diversity and emphasised the need for African-led pharmacogenomic research before population-specific clinical recommendations can be implemented [138].

#### 4.4.2. Need for Pharmacogenetic Testing

The lack of data on chemotherapy-induced toxicities and relative dose intensity in sub-Saharan Africa represents a significant knowledge gap. Implementing pharmacogenetic testing to identify patients at high risk for toxicity could enable personalised dosing strategies, improving both safety and efficacy. However, pharmacogenetic testing infrastructure is limited across Africa, and the cost-effectiveness of testing in resource-limited settings requires evaluation.

## 5. Discussion

Breast cancer management in Africa is at an inflexion point. This review has synthesised evidence spanning screening and early detection, diagnostic imaging, tissue and molecular diagnostics, AI-assisted technologies, and therapeutic management. Across these domains, a consistent picture emerges: although significant scientific and technological advances are available globally, their translation into clinical benefit for African women remains constrained by structural, biological, and evidence-based barriers.

The literature broadly supports the view that population-based mammography screening, which remains a cornerstone of early detection in many high-income countries, is not currently feasible or biologically appropriate as a primary screening modality across most of sub-Saharan Africa. The younger age distribution of African breast cancer patients reduces mammographic sensitivity because of the greater prevalence of dense breast tissue, while economic and infrastructural constraints limit the feasibility of large-scale mammography programmes in many healthcare systems.

Clinical breast examination has demonstrated value for clinical downstaging, with evidence suggesting a 17 to 47% shift from late- to early-stage diagnosis in programme settings. However, important questions remain regarding the optimal screening intervals and eligible age ranges for CBE in African populations, where breast cancer frequently presents at younger ages. In addition, variability in CBE performance between operators and healthcare settings limits its reliability as a standalone approach, while standardised training protocols validated for African healthcare workforces remain limited. Establishing the minimum requirements for effective CBE implementation, including appropriate training, referral pathways, and quality assurance, therefore remains an important research and policy priority.

Mobile mammography through NGO-led programmes represents an important equity-driven innovation, particularly in settings where access to conventional screening services is limited. However, the literature highlights a critical structural limitation: screening in isolation is unlikely to improve outcomes when diagnostic confirmation and treatment are not readily accessible. Concerns raised regarding the quality and clinical integration of NGO-led mobile screening services have not yet been adequately addressed through prospective studies incorporating meaningful clinical or survival endpoints.

Ultrasound, and particularly point-of-care ultrasound, is increasingly supported as a potentially appropriate and accessible imaging modality for African settings, given its utility in dense breast tissue and lower infrastructure requirements and cost relative to mammography. However, the absence of formal consensus within international guidelines regarding ultrasound as a primary screening tool creates uncertainty around its incorporation into national breast health strategies.

### 5.1. Diagnostic and Molecular Challenges

The literature reviewed also raises important questions regarding the interpretation of breast cancer biology in African populations. In particular, the widely reported predominance of triple-negative breast cancer among African women may be substantially influenced by pre-analytical and analytical variability in tissue handling and immunohistochemistry. Inaccurate receptor classification may result in hormone receptor-positive patients being denied effective endocrine therapy, while also contributing to an overestimation of the biological distinctiveness of breast cancer in African populations.

IHC therefore remains an indispensable component of molecular subtyping in African settings, but its implementation is undermined by power instability, reagent supply constraints, non-standardised fixation practices, and limited access to external quality assurance programmes. Although there is broad agreement that standardisation, quality assurance, and investment in anatomical pathology capacity are required, there remains limited evidence regarding how these interventions can be implemented sustainably at scale across diverse public-sector healthcare systems.

The emergence of mRNA-based alternatives, such as the Xpert STRAT4 assay, represents a promising approach, particularly because of its high concordance with IHC and its potential to leverage existing GeneXpert infrastructure. However, broader cost-effectiveness assessments and implementation studies across different African healthcare settings are required before these technologies can be confidently incorporated into national diagnostic algorithms.

Liquid biopsy similarly occupies a promising but relatively immature position within the African diagnostic landscape. Its potential advantages are particularly relevant to the continent, including non-invasive sample collection, opportunities for real-time disease monitoring, and reduced dependence on repeated tissue sampling for patients who may travel considerable distances to access specialised care.

### 5.2. Artificial Intelligence and Emerging Technologies

Across the AI literature reviewed, AI-assisted technologies for imaging interpretation, pathology image analysis, and treatment triage demonstrate considerable potential to address shortages of radiologists and pathologists in sub-Saharan Africa. Proof-of-concept studies from South Africa, Rwanda, Ethiopia, Kenya, and Burkina Faso indicate that AI-augmented workflows can extend the diagnostic reach of limited specialist expertise, reduce unnecessary referrals, and achieve acceptable accuracy within defined operational contexts.

However, an important limitation is common across these studies: there are currently no validated breast cancer imaging or pathology models trained predominantly or exclusively on sub-Saharan African datasets. This raises concerns regarding the generalisability of model performance to populations with different breast density profiles, tumour phenotype distributions, histological characteristics, and healthcare workflows.

Future efforts should prioritise the establishment of large, expert-labelled African breast imaging and pathology datasets and their development as accessible resources to support African-led AI research.

### 5.3. Therapeutic Management and Access

The evidence reviewed across systemic therapy modalities reveals a consistent tension: the global evidence base supporting effective breast cancer treatment is extensive, but access to these treatments and the infrastructure required to deliver them remains severely constrained for many African patients.

Chemotherapy, particularly anthracycline- and taxane-based regimens, remains among the most widely accessible systemic treatments and forms an important component of care for TNBC, locally advanced disease, and high-risk early-stage disease. Treatment completion rates as low as 48% in some Nigerian cohorts illustrate that nominal drug availability does not necessarily translate into effective treatment delivery. Supply chain disruptions, financial toxicity, inadequate supportive care, and insufficient toxicity monitoring can collectively undermine outcomes even when chemotherapy is technically available. Strengthening treatment-delivery infrastructure should therefore be considered alongside efforts to expand drug availability.

For endocrine therapy, tamoxifen availability has improved considerably; however, a 72% hormone-receptor-positive patient completion rate in South Africa, despite its relatively more developed healthcare infrastructure, illustrates the gap between treatment initiation and sustained treatment delivery. Furthermore, underdiagnosis of hormone receptor positivity resulting from inadequate IHC quality may further reduce the number of patients who are correctly identified, initiated on, and ultimately complete endocrine therapy.

HER2-targeted therapies, PARP inhibitors, and antibody–drug conjugates represent important advances in precision oncology but remain largely inaccessible across much of Africa. High acquisition costs, limited companion diagnostic infrastructure, and inadequate reimbursement mechanisms are major barriers.

Radiotherapy represents another major structural bottleneck, with consequences that extend beyond radiotherapy itself. The documented preference for mastectomy over breast-conserving surgery when access to postoperative radiotherapy is uncertain illustrates how limitations in one component of the care pathway can directly influence clinical decision-making elsewhere. The adoption of hypofractionated radiotherapy schedules, which reduce treatment duration without compromising outcomes, offers a pragmatic opportunity to improve throughput within constrained radiotherapy services and is broadly supported by the available evidence.

### 5.4. Pharmacogenomics and Precision Oncology

Perhaps the most significant knowledge gap identified across this review lies within pharmacogenomics. African populations harbour distinct pharmacogenetic variations, including variants in CYP2D6, CYP2J2, and DPYD, among others, that may influence drug metabolism, toxicity, and treatment efficacy. The potential clinical implications are substantial.

Population-specific pharmacogenomic data from African cohorts are therefore urgently required. African-led studies that are prospective, adequately powered, and linked to clinical outcome data will be essential for establishing the evidence base required to develop safe and equitable precision-dosing strategies.

### 5.5. Cross-Cutting Priorities for African Breast Cancer Research

Several cross-cutting themes emerge from the evidence synthesised in this review. First, the most pervasive knowledge gap is the underrepresentation of African populations—not only biologically and genomically, but also clinically and geographically—in the evidence base informing global breast cancer practice. Addressing this disparity requires more than increasing research capacity. It requires African-led research programmes in which study designs, research questions, and implementation frameworks are developed in response to African healthcare realities rather than primarily adapted from high-income settings.

Second, financial toxicity and health-system delays should not be considered peripheral to breast cancer management. The evidence reviewed demonstrates that diagnostic accuracy, treatment delivery, and ultimately patient outcomes are influenced by whether patients can afford to complete the care pathway and how efficiently healthcare systems move patients between its different stages. Future intervention research should therefore integrate health economics, implementation science, and patient-centred outcomes alongside conventional clinical and biological endpoints.

Third, multi-omics and systems biology approaches offer considerable potential for investigating the biological determinants of breast cancer in African populations, including differences in TNBC prevalence, age at diagnosis, and genomic characteristics. However, the successful implementation of these approaches requires large, harmonised African biobanks with comprehensive clinical annotation. Such infrastructure remains limited and geographically concentrated. Establishing well-governed biobanks linked to clinical databases would therefore provide a foundational resource capable of supporting research across diagnostics, therapeutics, pharmacogenomics, and tumour biology.

Finally, the integration of AI, multi-omics, liquid biopsy, and pharmacogenomics will only translate into meaningful clinical benefit if these technologies are developed and validated using data that adequately represent African populations. The evidence reviewed makes this requirement increasingly clear. The next step is to translate this recognition into sustained investment, collaborative infrastructure, and African research leadership capable of generating the evidence needed to guide implementation. Ultimately, advancing breast cancer care across Africa will require not simply the adoption of emerging technologies, but the development of African-relevant evidence, infrastructure, and implementation strategies that allow these technologies to be applied equitably and effectively.

## 6. Conclusions

Breast cancer care in Africa is at a critical juncture, with rapid advances in biology, diagnostics, artificial intelligence, and precision therapeutics offering new opportunities to improve patient outcomes. However, the translation of these advances into meaningful clinical benefit remains limited by the underrepresentation of African populations in research, constrained healthcare infrastructure, and substantial disparities in access to diagnosis and treatment. Addressing these challenges will require investment not only in emerging technologies, but also in African-led research, representative datasets and biobanks, diagnostic and treatment infrastructure, and context-specific implementation strategies. Integrating these priorities with advances in multi-omics, AI, pharmacogenomics, and precision oncology may enable more biologically informed and equitable breast cancer care across the continent. Ultimately, ensuring that future advances are developed with African populations, rather than simply applied to them, will be essential to reducing disparities and improving breast cancer outcomes in Africa.

## Figures and Tables

**Table 1:** Advances of AI technologies in Africa.

Country	AI Tool	Stage/Setting	Reference
South Africa	Breast AI	Daspoort PoliClinic, Gauteng	[35,39]
Rwanda	AI-Supported breast ultrasound	Butaro District Hospital partnered with Boston-based radiologists	[81,86]
Kenya	MOMMOAI	Teaching hospital pilot; trained on Saudi + Kenyan data	[85]
Ghana	Cross-regional fairness validation	Appiah study comparing Ghana/UK/Portugal/Poland datasets	[87]
Nigeria	Hurone AI	Multi-hospital AI oncology platforms	[88]
Uganda	African Breast Imaging Dataset (open source)	ECUREI/MAI lab collaboration	[49]
Uganda + Nigeria	Open Africa Breast ultrasound + mammogram dataset initiative	Kawooya et al. longitudinal dataset	[49]
Pan-Africa	DataPathology, PapsAI, MinoHealth	Pathology AI innovations, multi-country rollouts	[88]

## Data Availability

No new data were created or analysed in this study. Data sharing is not applicable to this article.

## Abbreviations

The following abbreviations are used in this manuscript:

ADC	Antibody-drug conjugate
AI	Artificial intelligence
ALND	Axillary lymph node dissection
BCS	Breast-conserving surgery
BI-RADS	Breast Imaging Reporting and Data System
BRCA1/2	Breast cancer susceptibility gene 1/2
CBE	Clinical breast examination
cfDNA	Cell-free DNA
CNB	Core needle biopsy
CTC	Circulating tumour cell
ctDNA	Circulating tumour DNA
CYP2D6	Cytochrome P450 2D6
CYP2J2	Cytochrome P450 2J2
DNA	Deoxyribonucleic acid
DPYD	Dihydropyrimidine dehydrogenase
ER	Oestrogen receptor
FFPE	Formalin-fixed paraffin-embedded
FNA	Fine needle aspiration
GP	General practitioner
HER2	Human epidermal growth factor receptor 2
HIC	High-income country
HIV	Human immunodeficiency virus
HR+	Hormone receptor-positive
IHC	Immunohistochemistry
Ki-67	Ki-67 proliferation marker
LINAC	Linear accelerator
LMIC	Low- and middle-income country
MDT	Multidisciplinary team
MRI	Magnetic resonance imaging
mRNA	Messenger RNA
NACT	Neoadjuvant chemotherapy
NCI	National Cancer Institute
NGO	Non-governmental organisation
NGS	Next-generation sequencing
PARP	Poly(ADP-ribose) polymerase
POCUS	Point-of-care ultrasonography
PPP	Public–private partnership
PPV	Positive predictive value
PR	Progesterone receptor
RDISA	Radhiant Diagnostic Imaging South Africa
SAHPRA	South African Health Products Regulatory Authority
SLNB	Sentinel lymph node biopsy
SSA	Sub-Saharan Africa
T-DM1	Trastuzumab emtansine
T-DXd	Trastuzumab deruxtecan
TB	Tuberculosis
TNBC	Triple-negative breast cancer
ULF	Ultra-low field
VEGFR2	Vascular endothelial growth factor receptor 2
WHO	World Health Organization
